# Noninvasive Assessment of Response to Neoadjuvant Chemotherapy in Osteosarcoma of Long Bones with Diffusion-Weighted Imaging: An Initial In Vivo Study

**DOI:** 10.1371/journal.pone.0072679

**Published:** 2013-08-26

**Authors:** Cheng-Sheng Wang, Lian-Jun Du, Ming-Jue Si, Qi-Hua Yin, Liang Chen, Min Shu, Fei Yuan, Xiao-Chun Fei, Xiao-Yi Ding

**Affiliations:** 1 Department of Radiology, Ruijin Hospital, Shanghai Jiao Tong University School of Medicine, Shanghai, China; 2 Department of Radiology, Union Hospital, Fujian Medical University, Fuzhou, China; 3 Department of Pathology, Ruijin Hospital, Shanghai Jiao Tong University School of Medicine, Shanghai, China; Johns Hopkins University, United States of America

## Abstract

**Objectives:**

The purpose of our study is to investigate whether diffusion-weighted imaging (DWI) is useful for monitoring the therapeutic response after neoadjuvant chemotherapy in osteosarcoma of long bones.

**Materials and methods:**

Conventional magnetic resonance imaging (MRI) and DWI were obtained from 35 patients with histologically proven osteosarcomas. MR examinations were performed in all patients before and after 4 courses of preoperative neoadjuvant chemotherapy. Apparent diffusion coefficients (ADC) were measured. The degree of tumor necrosis was assessed macroscopically and histologically by two experienced pathologists after operation. Student’s *t* test was performed for testing changes in ADC value. Pearson’s correlation coefficient was used to estimate the correlation between necrosis rate and post- neoadjuvant chemotherapy ADC values. *P*<0.05 was considered to denote a significant difference.

**Results:**

The difference of the whole osteosarcoma between pre- neoadjuvant chemotherapy ADC value (1.24±0.17×10^−3^ mm^2^/s) and post- (1.93±0.39×10^−3^ mm^2^/s) was significant difference (*P*<0.01). Regarding in patients with good response, the post- neoadjuvant chemotherapy values were significantly higher than the pre- neoadjuvant chemotherapy values (*P*<0.01). The post- neoadjuvant chemotherapy ADC value in patients with good response was higher than that of poor response (*t* = 8.995, *P*<0.01). The differences in post- neoadjuvant chemotherapy ADC between viable (1.03±0.17×10^−3^ mm^2^/s) and necrotic (2.38±0.25×10^−3^ mm^2^/s) tumor was highly significant (*t* = 23.905, *P*<0.01). A positive correlation between necrosis rates and the whole tumor ADC values (*r* = 0.769, *P*<0.01) was noted, but necrosis rates were not correlated with the ADC values of necrotic (*r* = −0.191, *P* = 0.272) and viable tumor areas (*r* = 0.292, *P* = 0.089).

**Conclusions:**

DWI can identify residual viable tumor tissues and tumor necrosis induced by neoadjuvant chemotherapy in osteosarcoma. The ADC value can directly reflect the degree of tumor necrosis, and it is useful to evaluate the preoperative neoadjuvant chemotherapy response in patients with osteosarcoma.

## Introduction

Osteosarcoma is the most common bone sarcoma and the third most common malignancy in children and adolescents [1]. A combination of surgery and chemotherapy seems to be the normal choice of treatment and chemotherapy may function better if used both preoperatively and postoperatively [2]. With adjuvant or neoadjuvant chemotherapy, the therapeutic efficacy of osteosarcoma has been greatly improved since 1970s [3], and five-year disease-free survival rates have been raised from 15%∼20% to 70%∼80%. Limb salvage rate is obviously increased [3], but there are still 20%∼30% of patients with poor curative effect. The extent of the histological response to chemotherapy evaluated at the time of surgery has been shown to be the most reliable prognostic factor in patients with localized lesion up to now. Treatment response is considered successful if more than 90% of the tumor cells show necrosis histologically [4].

In clinical practice, the patients’ subjective response, clinical examination and radiographic changes cannot accurately and quantitatively reflect the efficacy of chemotherapy. It is necessary to find an effective method to evaluate the curative effect of neoadjuvant chemotherapy, which has great importance for surgical planning, postoperative chemotherapy selection and prognostic judgement. Diffusion-weighted imaging (DWI) assesses response to chemotherapy which manifests itself as a change in cellularity within tumor using water diffusion [5]. In osteosarcoma, DWI is correlated directly with tumor necrosis [4]. During chemotherapy of osteosarcomas, tumor ADC changes are related to the degree of tumor necrosis [5]. Chemotherapy response can be predicted and evaluated by DWI early in the disease course and it correlates well with necrosis [6]. Accordingly, the ADC value on DWI is a promising tool for monitoring the therapeutic response of primary bone sarcomas [7]. To the best of our knowledge, previous studies delineating the therapeutic response of osteosarcoma by DWI and the number of patients or animal models were both extremely limited [4]–[9]. In addition, the patients’ baseline and chemotherapy scheme were differ.

DWI is used to measure treatment response, but it has not been extensively explored if it is having some correlation with necrosis rate and can be used to differentiate between viable and necrotic tumor areas. What is more, a relatively large sample study with unified clinical stage and chemotherapy scheme is necessary. With this background we designed a study to study the correlation between baseline and post- neoadjuvant chemotherapy in 35 patients with osteosarcoma as detected by DWI and pathological examination.

## Materials and Methods

### Ethics Statement

Approval to undertake this study was granted by the Ethics Review Committee of Ruijin Hospital, Shanghai Jiao Tong University School of Medicine and was conducted according to the Declaration of Helsinki Principles. Written informed consent from all adult participants or guardians of the minors/children participants was obtained.

### Participants

Therapy-naive osteosarcoma patients with adequate organ function for receiving neoadjuvant chemotherapy were qualified for the study. Patients with other concurrent systematic diseases that would harm the safety of the patient or the patient’s ability to complete the study were excluded. Thirty-five patients with osteosarcoma in long bone were recruited for this study. There were 18 men and 17 women, with a mean age at diagnosis of 26.8 (7–65) years. The vast majority of tumors were located in the metaphysis of the long bones. Nineteen were located in the distal femur, followed by proximal tibia (n = 11), proximal femur (n = 2), distal tibia (n = 1), proximal fibula (n = 1) and proximal humerus (n = 1). Enneking stage system [10] for all tumors were IIA–IIB. The duration of symptoms ranged from 2 weeks to 6 months, with a mean of 2.5 months. The main symptoms were local swelling/mass, pain and limitation of activity, of which 12 cases with local swelling/mass and pain, 10 cases with pain and limitation of activity, 7 cases only with local swelling/mass and 6 cases only with pain as the initial symptoms. Written informed consent was obtained from each patient. The study was approved by the institutional review board.

### Neoadjuvant Chemotherapy and Imaging Procedures

Thirty patients were performed neoadjuvant chemotherapy regimens of cisplatin+methopterin+epirubicin+iphosphamide, and 5 cases individualized intermittent neoadjuvant chemotherapy regimens of paclitaxel+cisplatin with folic acid for detoxification.

Two MR examinations were performed as follow: the first before CT-guided or open biopsy, and the second after 4 courses of preoperative neoadjuvant chemotherapy. MRI was performed on a 1.5-T Signa HD MR system (General Electric Medical Systems, Milwaukee, WI, USA) using our routine protocol for evaluating the tumor. An appropriate surface or body coil was used. A combination of axial, sagittal, and coronal images was obtained using T1-weighted spin-echo sequence (TR range/TE range, 360–590/13–21; matrix size, 256×256), T2-weighted fast spin-echo sequence (TR range/TE range, 3000–5180/62–101; matrix size, 256×256) and STIR sequence (TR range/TE range/TI range, 3000–5000/53–91/150–170; matrix size, 256×256).

DWI was performed in the axial plane using a single shot echo planar imaging (SS-EPI) sequence with the following parameters: TR/TE = 3000 ms/95 ms, 220 mm FOV, 128×64 pixel matrix size, 5-mm section thickness, 1-mm interslice gap, and number of excitation, 6. DWI were acquired with diffusion gradient encoding in 3 orthogonal directions with b values (0 and 700 s/mm^2^). In all images, a fat-saturated pulse was used to exclude chemical-shift artifacts.

Contrast-enhanced images of axial, sagittal and coronal plane were obtained using a T1-weighted spin-echo sequence with and without fat suppression (TR range/TE range, 360–590/13–21; matrix size, 256×256) after the injection of 0.1 mmol/kg of body weight of gadopentetate dimeglumine (Magnevist; Schering [now Bayer Health-Care]) injected at 2 ml/s, followed by a 20 ml normal saline flush.

### Diameter Measurement and Volume Calculation of Tumor

Three-dimensional tumor diameters of each patient were measured on T2-weighted images on the basis of the description by Bieling et al. [11]. Tumor volume (TV) was calculated from the largest tumor length (TL), tumor width (TW), and tumor depth (TD). According to the method described by Gobel et al [12], tumor volumes of ellipsoidal tumors with soft tissue extension were calculated using the formula TV = 0.53×TL×TW×TD; tumor volumes of cylindric tumors without soft tissue extension were calculated using the formula TV = 0.785×TL×TW×TD.

### Image Postprocessing

The raw data were transferred to a computer workstation (Sun Microsystems, ADW4.2), where the DWI data was processed using Functool 2 image analysis software (GE Medical Systems, Milwaukee, Wisc., USA) to obtain the pre- and post-treatment tumor apparent diffusion coefficient (ADC) maps and ADC histograms. The ADC values were obtained in an automated way by measuring the intensity of the ADC maps. The region of interests (ROIs) of the solid area, necrotic area and whole lesion chosen for ADC measurement were based on standard MR images both with and without contrast-enhancement and DW images. The difference between the features extracted from pre- and post-treatment tumor ADC histograms were also observed.

### Tissue Processing and Pathological Assessment

**Figure 1:**
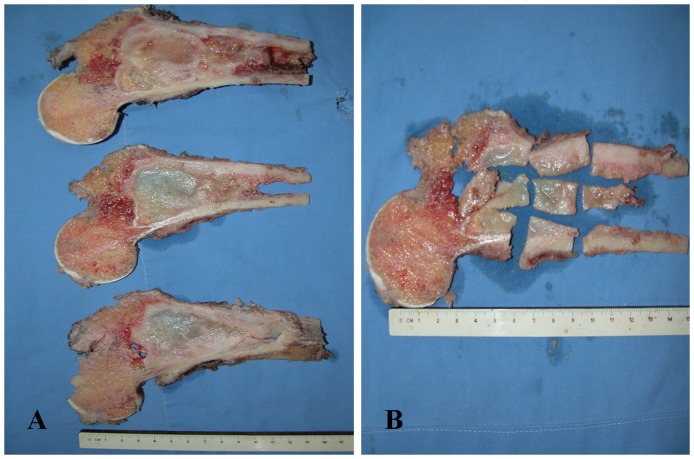
Tissue processing of the resected specimen. (**A**) Samples were collected from the center of the resected tumor. (**B**) After dividing the tissue into small pieces, all small pieces of tissues were embedded and numbered. Necrosis rate was determined.

After neoadjuvant chemotherapy, all patients underwent limb salvage surgery or amputation within 2 weeks. The resected tumor was photographed. Axial, coronal or sagittal sections were made (Figure 1A). After dividing the whole tumor tissue into small pieces, all small pieces of tissues were embedded and numbered so as to reappear their exact locations (Figure 1B). All tumor tissues were analyzed macroscopically and histologically for the degree of necrosis. The effectiveness of chemotherapy was defined as “good” (≥90% tumor necrosis) or “poor” (<90% tumor necrosis) [6], [7], [13]. All tumor specimens were examined by two experienced pathologists to determine the percentage of tumor necrosis. In the event of disagreement, a consensus was arrived at by discussion.

### Statistical Analysis

SPSS version 13.0 (SPSS, Chicago, IL) was used for data analysis. ADC values and necrosis rates were calculated along with 95% confidence intervals. The differences in the tumor size and volume, ADC values between the pre- and post- neoadjuvant chemotherapy, the post- neoadjuvant chemotherapy ADC values between viable and necrotic tumor areas, were compared using the paired *t* test. Regarding the ADC values pre- and post- neoadjuvant chemotherapy, the differences between patients with good and poor response were compared using the independent-samples *t* test. Pearson’s correlation coefficient was used to estimate the correlation between necrosis rate and post- neoadjuvant chemotherapy ADC values. For all analyses, *P*<0.05 was considered to denote a significant difference.

## Results

### Tumor Size and Volume at Pre- and Post- Neoadjuvant Chemotherapy

The mean values of tumor size and volume as obtained in the MRI pre- and post- neoadjuvant chemotherapy are shown in Table 1. These two groups were similar with respect to maximum dimension, intramedullary extension and tumor volume (*P*>0.05).

**Table 1:** Tumor size and volume at pre- and post-chemotherapy.

Variable	Pre-chemotherapy	Post-chemotherapy	*t*	*P*
Maximum dimension (cm)	11.06±3.30	10.21±2.93	1.202	0.237
Intramedullary extension (cm)	10.50±2.33	9.62±1.22	1.860	0.072
Volume (ml)	148.2±60.6	156.8±75.3	1.595	0.120

### Pathological Assessment and Operation Type

On pathological evaluation, all patients showed different degrees of necrosis, of which 12 patients with poor response and the rest 23 good response. The percentage of necrosis rates were 50.2±24.3 (95% CI: 31.6–68.9) and 94.2±2.5 (95% CI: 93.2–95.2) respectively. All 35 patients underwent surgical intervention; limb salvage surgery could be performed in 26 patients while 9 patients underwent amputation.

### Mean ADC Values at Pre- and Post- Neoadjuvant Chemotherapy

**Figure 2:**
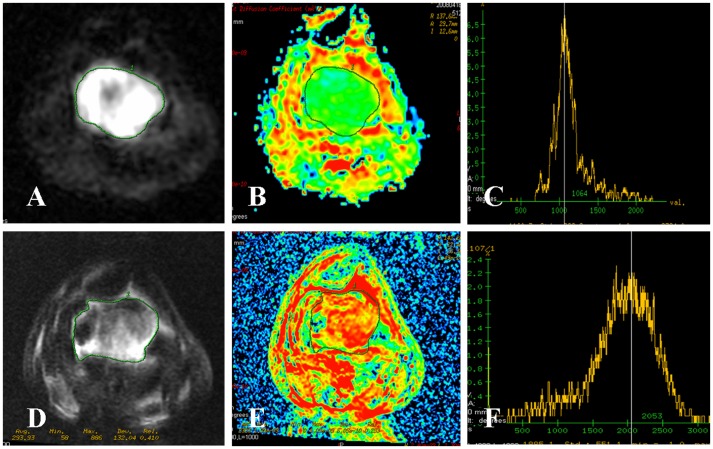
Osteosarcoma of the distal femur in a 24-year-old man with good response. (**A∼C**) DWI map, ADC map and ADC histogram before neoadjuvant chemotherapy. The signal intensity of tumor on DWI map was high. The ADC value of the whole tumor was 1.12×10^−3^ mm^2^/s with a green area. The ADC histogram was high and sharp. (**D∼F**) DWI map, ADC map and ADC histogram after neoadjuvant chemotherapy. The signal intensity of tumor on DWI map was decreased. The ADC value of the whole tumor was increased to 1.99×10^−3^ mm^2^/s with a subtotal red area. The ADC histogram was short, wide and moving to the right of the coordinate. Tumor necrosis rate of 92% was confirmed by postoperative pathological evaluation. The effectiveness of neoadjuvant chemotherapy was good.

**Figure 3:**
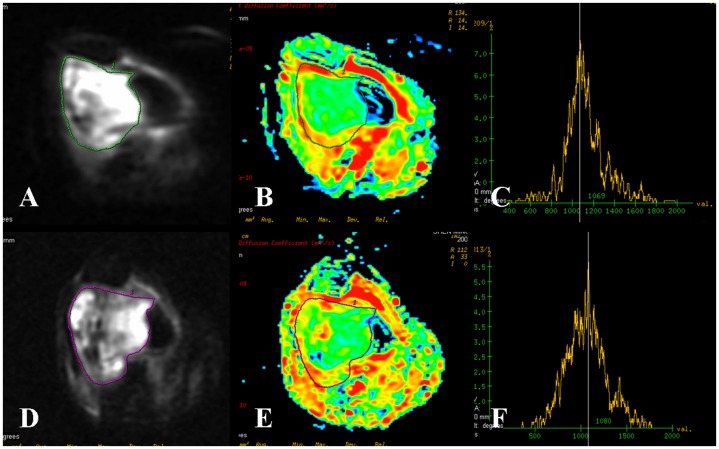
Osteosarcoma of the distal femur in a 46-year-old woman with poor response. (**A∼C**) DWI map, ADC map and ADC histogram before neoadjuvant chemotherapy. The tumor on DWI map was mixed high signal intensity. The ADC value of the whole tumor was 1.35×10^−3^ mm^2^/s with a subtotal green area. The ADC histogram was high and sharp. (**D∼F**) DWI map, ADC map and ADC histogram after neoadjuvant chemotherapy. The tumor on DWI map was mixed signal intensity. The ADC value of the whole tumor (1.31×10^−3^ mm^2^/s) and the ADC histogram were similar to those of pre- neoadjuvant chemotherapy. Tumor necrosis rate of 20% was confirmed by postoperative pathological evaluation. The effectiveness of neoadjuvant chemotherapy was poor.

The mean ADC values of various variables as obtained in the MRI pre- and post- neoadjuvant chemotherapy are shown in Table 2. In our study, the mean pre- neoadjuvant chemotherapy ADC value of the whole osteosarcoma was 1.24±0.17 (95% CI: 1.18–1.30)×10^−3^ mm^2^/s and post- neoadjuvant chemotherapy it was 1.93±0.39 (95% CI: 1.80–2.07)×10^−3^ mm^2^/s. The mean ADC values of the whole tumors before and after neoadjuvant chemotherapy in patients with good response were 1.21±0.17 (95% CI: 1.14–1.28)×10^−3^ mm^2^/s and 2.18±0.16 (95% CI: 2.11–2.25) ×10^−3^ mm^2^/s respectively, while in patients with poor response were 1.31±0.16 (95% CI: 1.21–1.41)×10^−3^ mm^2^/s and 1.47±0.25 (95% CI: 1.31–1.62)×10^−3^ mm^2^/s respectively. Regarding in patients with good response, the post- neoadjuvant chemotherapy value was significantly higher than the pre- neoadjuvant chemotherapy values (*P*<0.01). The ADC histogram showed different degrees of short and wide from initial high and sharp, and moving to the right of the coordinate (Figure 2A∼F). In patients with poor response, the ADC values between pre- and post- neoadjuvant chemotherapy were not significant difference (*P*>0.05). The post- neoadjuvant chemotherapy ADC histogram was similar to that of pre- or with a little change (Figure 3A∼F). The post- neoadjuvant chemotherapy ADC value in patients with good response was higher than that of poor response (*t* = 8.995, *P*<0.01), and the pre- neoadjuvant chemotherapy values were not statistically significant between patients with good response and poor response (*t* = 1.699, *P* = 0.099).

**Table 2:** Mean ADC values of whole tumor at pre- and post-chemotherapy (×10^−3^ mm^2^/s).

Variable	Pre-chemotherapy	Post-chemotherapy	*t*	*P*
All patients	1.24±0.17	1.93±0.39	8.389	0.000
Patients with good response	1.21±0.17	2.18±0.16	15.013	0.000
Patients with poor response	1.31±0.16	1.47±0.25	2.090	0.061

Post- neoadjuvant chemotherapy necrotic tumor areas, which were confirmed by pathological examination, showed mean ADC value was 2.38±0.25 (95% CI: 2.30–2.47)×10^−3^ mm^2^/s. Viable tumor areas revealed lower ADC value [mean 1.03±0.17 (95% CI: 0.97–1.09)×10^−3^ mm^2^/s]. The difference in ADC between viable and necrotic tumor areas was highly significant (*t* = 23.905, *P*<0.01).

### Correlation Analysis of Necrosis Rate with Post- Neoadjuvant Chemotherapy ADC Values

**Figure 4:**
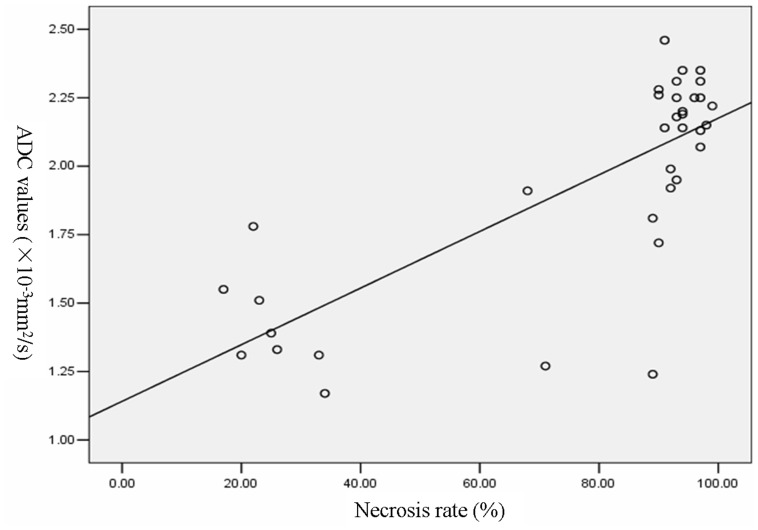
Correlation of ADC values of the whole osteosarcomas with necrosis rates after neoadjuvant chemotherapy.

Pearson’s correlation analysis was applied in the post- neoadjuvant chemotherapy ADC values of necrotic tumor areas, viable tumor areas, as well as the whole tumor in order to calculate the strength of the relationship with necrosis rate. A positive correlation between necrosis rate and the whole tumor (*r* = 0.769, *P*<0.01) was noted (Figure 4), but necrosis rate was not correlated with the necrotic (*r* = −0.191, *P* = 0.272) and viable tumor areas (*r* = 0.292, *P* = 0.089).

## Discussion

For patients with osteosarcoma, monitoring the curative effect of chemotherapy or radiotherapy is very important for surgical planning and postoperative chemotherapy selection. The current gold standard for judging the curative effect of chemotherapy is tumor necrosis rate using histological method, but this method is traumatic due to repeated biopsy [5], [9]. The possibility of using specified tumor-adapted therapies and the use of novel anticarcinogenic treatments further support the demand for noninvasive imaging modalities. DWI is currently the only imaging method for non-invasively measuring the local diffusion characteristics of water molecules *in vivo*. It can reflect the information of spatial composition and the functional status of water exchange among various tissues in pathophysiological states from the molecular level. Accordingly, DWI can detect the early changes of morphology and pathology related to water content of tissue [14], and ADC value can reflect the degrees of tissue water diffusion.

In present study, tumor size and volume as determined by MR showed no significant difference between pre- and post- neoadjuvant chemotherapy. These may be explained by the intraosseous component remains almost constant and it is only the extraosseous component that changes [15]. The slow regression of osteoid matrix and cystic degeneration in good responders with corresponding illegitimate increase in size on MRI may contribute the results as well [16].

In contrast to the measurement of tumor size and volume, ADC-measurements were shown to be superior to monitor treatment efficacy. DWI is a sensitive imaging modality capable of detecting early cellular changes in therapeutic tumors, which precede macroscopical voluminal response [17], [18]. The post- neoadjuvant chemotherapy ADC values were significantly higher than the pre- neoadjuvant chemotherapy values both in patients with good response and poor response. The post- neoadjuvant chemotherapy ADC value in patients with good response was increased significantly. The ADC histogram showed different degrees of short, wide and moving to the right of the coordinate after neoadjuvant chemotherapy. The mechanism of action of cytotoxic chemotherapeutic drugs is to promote cell death through destroying cell membrane integrity and increasing cell membrane permeability. The water mobility within a tumor will increase over time after treatment and that the magnitude of change would be related to the effectiveness of therapy, which results in subsequent reduction in cell density [4].

The results also showed that the ADC values were significant difference between viable and necrotic tumor areas after neoadjuvant chemotherapy. The mean ADC value of post- neoadjuvant chemotherapy necrotic tumor areas was 2.38±0.25×10^−3^ mm^2^/s, and viable tumor areas 1.03±0.17×10^−3^ mm^2^/s. The reason is the difference in cell density, cell membrane integrity, as well as molecular content of free water between viable and necrotic tumor areas. The determination of ADC value by DWI can distinguish viable and necrotic tumor areas, and then noninvasively, dynamically, early detect curative effect of chemotherapy in malignant tumor [5], [9].

Change in ADC of whole tumor correlates well with response to neoadjuvant chemotherapy in different necrosis rates, which could be detected before morphologic changes indicated a significant difference in the tumor response. DWI was correlated directly with tumor necrosis in present study. According to these preliminary results, calculation of an ADC value seems to be a promising quantitative and sensitive surrogate to monitor response to chemotherapy in osteosarcomas.

The limitations of our study should be considered. First, heterogeneity is obvious in osteosarcoma due to different histological subtypes, especially the pathological changes are complicated after neoadjuvant chemotherapy. The neoplastic bone, cartilage, calcification and residual cortical bone can decrease the ADC value. Second, all patients were examined before and after neoadjuvant chemotherapy. It was impossible to observe the change of ADC value dynamically at difffernt time courses. Third, tumor volume was calculated by simple mathematical formulas. An irregular shape might not fit well into an ellipsoidal or cylindric formula, and there is a risk of over- or underestimation of tumor volume. Fourth, we correlated the MR images with the specimens location by location, but some errors may unavoidable due to mismatch among slices.

To sum up, as an imaging modality to detect the microscopic diffusion activities of water molecules *in vivo*, DWI reflects the microstructure of tissue from the cellular and molecular levels which can identify residual viable tumor tissues and tumor necrosis induced by neoadjuvant chemotherapy. The ADC value can directly reflect the degree of tumor necrosis, and it is useful to evaluate the curative effect of neoadjuvant chemotherapy promptly and objectively.
